# A novel core-shell lipid nanoparticle for improving oral administration of water soluble chemotherapeutic agents: inhibited intestinal hydrolysis and enhanced lymphatic absorption

**DOI:** 10.1080/10717544.2017.1386730

**Published:** 2017-10-13

**Authors:** Tao Wang, Liao Shen, Zhen Zhang, Haiyan Li, Ri Huang, Yadan Zhang, Dongqin Quan

**Affiliations:** Beijing Institute of Pharmacology and Toxicology, Beijing, PR China

**Keywords:** Core-shell lipid nanoparticle, topotecan, intestinal lymphatic transportation, oral administration, water-soluble chemotherapeutical agents

## Abstract

The oral administration of water-soluble chemotherapeutical agents is limited by their serious gastrointestinal side effects, instability at intestinal pH, and poor absorption. Aiming to solve these problems, we chose topotecan (TPT) as a model drug and developed a novel lipid formulation containing core-shell lipid nanoparticle (CLN) that makes the water-soluble drug to ‘dissolve’ in oil. TPT molecules can be encapsulated into nanoparticles surrounded by oil barrier while avoiding the direct contact with intestinal environment, thus easing the intestinal hydrolytic degradation and gastrointestinal (GI) irritation. Microstructure and mean particle size of TPT-CLN were characterized by Transmission Electron Microscope (TEM) and Dynamic Light Scattering (DLS), respectively. The average size of nanoparticles was approximately 60 nm with a homogeneous distribution in shapes of spheres or ellipsoid. According to *in vitro* stability studies, more initial form of TPT was observed in presence of lipid nanoparticle compared with free topotecan solution in artificial intestinal juice (pH 6.5). After oral administration of TPT-CLN in rats, AUC and C_max_ of TPT were all increased compared with free TPT, indicating significant enhancement of oral absorption. Intestinal lymphatic transport was confirmed as the major way for CLN to enhance oral absorption of TPT by the treatment of blocking chylomicron flow. Lower GI irritation of TPT-CLN was observed in the gastrointestinal damage studies. The *in vivo* antitumor activity of TPT-CLN showed an improved antitumor efficacy by oral treatment of TPT-CLN compared to free TPT. From the obtained data, the systems appear an attractive progress in oral administration of topotecan.

## 1. Introduction

**Scheme 1.**
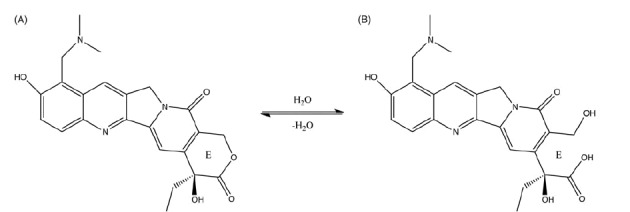
The hydrolysis of topotecan (A) to produce carboxylate (B).

Topotecan (TPT) is a water-soluble chemotherapeutic agent semi-synthesizing from camptothecin (CPT) (Sawada et al., 1991). As an inhibitor of topoisomerase I, TPT stabilizes the covalent complex between topoisomerase I and DNA, resulting in enzyme linked DNA cleavage and single strand breaks (Hsiang et al., 1985; Giovanella et al., 1989; Giovanella et al., 1991; Emerson et al., 1995). The first commercial product, Hycamtin^®^, as a dosage of intravenous injection, was approved by the Food and Drug Administration (FDA) in 1996 for the treatment of ovarian cancer and small cell lung cancer (Herben et al., 1996). Despite the marked antitumor efficacy, the dosage of Hycamtin^®^ may result in patient inconvenience and instantaneous hyper concentration in blood that is usually related to severe hematologic toxicity (Ozols, 2000; Saltz et al., 2001). In contrast, an oral formulation allows home-based drug intake which might be more patient-convenient and provide a lower systemic exposure profile with less fluctuation leading to lower grade IV neutropenia. Numerous studies confirmed that oral TPT appears promising in treatment of and small-cell lung cancers (Nicum & O’Brien, 2007; Wethington et al., 2008; Hagmann et al., 2015; Bruchim et al., 2016). However, Hydrolysis of the lactone ring of TPT, which forms more polar carboxylate with no biological activity, is known to occur if the pH of the solution rises above pH 4 (Scheme 1) (Burke, 1996; Giovanella et al., 2000; Padhi et al., 2016). Therefore, at physiological pH in intestinal tract, intensive hydrolytic degradation of TPT will lead to poor bioavailability and anti-tumor efficacy.

The intestinal lymph provides a specialized pathway for highly lipophilic xenobiotics to access to the systemic circulation (Qin et al., 2013). Hence, it can also provide a potential pathway of absorption for oral drug (Dahan & Hoffman, 2005). Lipid formulation could enhance oral bioavailability of drugs via lymphatic transport and segregate them from the aqueous environment resulting in more chemical stability (Trevaskis et al., 2008). However, to entrap the drug into lipid-based system was considered as the biggest difficulty in the development of lipid formulation for water-soluble substances such as TPT.

**Scheme 2.**
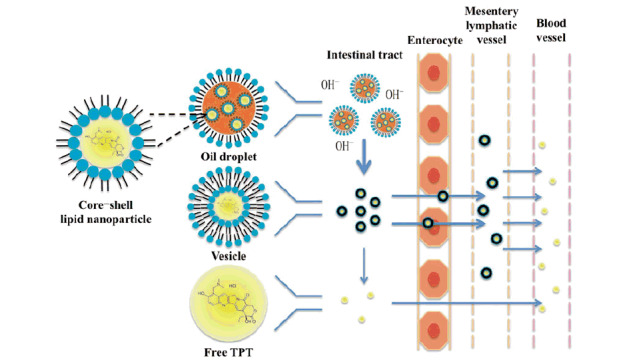
The mechanism of CLN system to inhibit hydrolysis and improve intestinal lymphatic transport while oral administrating.

Previously, we described a technique to encapsulate water-soluble drugs into oil solution by forming core-shell lipid nanoparticles (CLN) (Zhang et al., 2013). Different from conventional nanocarriers, liquid oil was used as dispersing medium in this system and phospholipids were used as the shell material of nanoparticles. Amphipathic phospholipid molecules can self-assemble together and regularly arrange a polar pool for hydrophilic molecules while the non-polar tails of phospholipid molecules radiating to the solvent. For oral administration, CLN could decrease the contact between drug molecules and intestinal juice to a certain extent, inhibit hydrolysis and increase their intestinal lymphatic transport thus a higher absorption and therapeutic efficacy can be achieved (Scheme 2).

The major objective of this study is to prepare CLN system encapsulating water-soluble drug, TPT and improve its stability, and oral bioavailability.

## 2. Materials and methods

### 2.1. Materials

1, 2-Dipalmitoyl-sn-glycero-3-phospho-rac-glycerol (DPPG-Na) and Soya Phosphatidyl Choline (LIPOID S100) were purchased from Lipoid GmbH (Friedenstrasse, Ludwigshafen, Germany); Cycloheximide was purchased from SERVA (Heidelberg, Baden-Württemberg, Germany). Topotecan Hydrochloride Capsules (XinZe^®^) was obtained from Rui Nian Best (Nan Jing, Jiangsu, China); active ingredient: Topotecan-HCl). Medium-chain triglycerides (MCTs) were purchased from Hunan ER-KANG Pharmaceutical Co., Ltd, Shenzhen, China. The glipizide was used as internal standard (IS) (National Institute for the Control of Pharmaceutical and Biological, Beijing, China). High-performance liquid chromatography (HPLC) grade Methanol was bought from Fisher Scientific (Fair Lawn, NJ). Wahaha (Hangzhou, China) deionized water was used throughout the study.

(S)-10-[(dimethylamino)methyl]-4-ethyl-4,9-dihydroxy-(H-Pyrano[3’,4’:6,7]indolizino[1,2-b]quinoline-3,14[4H,12H]-dionemonohydrochloride (Topotecan Hydrochloride, TPT) was supplied by SanHuan Chemistry (WuHan, China) and other chemicals used without further purification were HPLC or analytical reagent grade purchased from local commercial suppliers.

### 2.2. Preparation of core-shell lipid nanoparticles (CLN)

First, the blank small unilamellar liposomes (SUVs) with negative charged were obtained from procedures as follows: dissolve the S100 and DPPG-Na (with ratio of 95:5, w/w) in 15 mL chloroform to obtain a transparent solution, then the sample was evaporated by a rotary evaporator following with the addition of pure water. This solution was sonicated by the probe-type sonicator at 200 W for 15 min and was filtered through 0.2 μm membrane (Supor, Shanghai, China). TPT solution (1 mg/mL) was prepared by dissolving TPT in deionized water. After mixing TPT solution and SUVs together (in a ratio of 2:1, v/v) with 3.0 mL in total, the mixture was filled into a 7 mL vials and was lyophilized by a freeze drier for 48 h (FD-1; Beijing SiHuan Technology Company, Beijing, China) to remove the water. The chamber pressure was maintained at 10 Pa during the drying process. Finally, TPT was encapsulated in CLN when the lyophilized dry cake was dissolved in proper amount of MCT. The resulting oil solution contained TPT with the concentration of 4 mg/mL (0.4% w/v).

### 2.3. Physiochemical characterization of CLN

#### 2.3.1. Size distribution

The mean diameter and size distribution of CLN were evaluated by dynamic light scattering (DLS) using Malvern Zetasizer ZS 90 (Malvern, United Kingdom).

#### 2.3.2. The observation of morphology

The morphology of particles was observed by the transmission electron microscope (TEM, JEM-1230, JEOL, Japan). Before the examination, the 200 μL *oil solution* of CLN was diluted by 5 mL *n*-Heptane. Then 10 μL of the sample was dropped on a copper grid with carbon film followed by addition of 10 μL phosphotungstic acid solution (PTA), the excess fluid was removed by filter paper.

### 2.4. *In vitro* stability

The *in vitro* stability studies were performed on CLN formulation in comparison with TPT aqueous solution in an amount corresponding to 2 mg of TPT. Samples were placed in 50 mL medium of fasted State Simulated Intestinal Fluid (pH 6.5) at constant conditions (100 rpm and 37.0 ± 0.1 °C). At predetermined time points (at 10, 20, 30, 45, 60, 90, and 120 min), 0.5 mL aliquots of medium were collected and were centrifuged at 15,000×*g* for 30 s to get supernatant. 20 μL supernatant was analyzed for both (opened carboxylate and ring closed lactone form of TPT) content by HPLC using a gradient method previously described. The HPLC analysis was conducted using a Shimadzu HPLC system equipped with a SIL-20AC Autosampler, double LC-20 A pumps, a controller and an SPD-M20A Detector (Shimadzu, Japan). Chromatographic separation was achieved at 55 °C using a CAPCELL PAK C18 MGII cartridge column (250 × 4.6 mm, 5 μm, Shimadzu) and the sample temperature was maintained at 4 °C. The mobile phase consisted of mobile phase ‘A’ (1% Triethyleneamine in water, pH 6.4 adjusted with glacial acetic acid) and mobile phase ‘B’ (100% acetonitrile). Each sample was run for 27 min at a flow rate of 1.0 mL/min using a gradient method, where the amount of organic phase was increased from 12 to 40% over 8 min. This method was able to detect ring opened carboxylate (eluted at 8 min) and ring closed lactone form (eluted at 11 min) of TPT in a single run. The detection wavelength was 360 nm (Sai et al., 2002). Meanwhile, 100 μL aliquots were diluted with 900 μL hydrochloric acid and 20 μL of it was analyzed for total TPT content by HPLC using an isocratic elution. The mobile phase was an 80:20 (v/v) mixture of 0.1% trifluoroacetic acid (TFA) and acetonitrile. The flow rate was 1.0 mL/min, the detection wavelength was 267 nm, and the column temperature was maintained at 25 °C. All HPLC methods were validated by procedures in Chinese Pharmacopoeia (ChP).

### 2.5. *In vivo* studies

#### 2.5.1. Intestinal lymphatic absorption

The study protocol was approved by the ethical committee of Beijing Institute of Pharmacology and Toxicology in accordance with Chinese laboratory animals-guideline of welfare and ethics. Twenty-four Wistar male rats (200–250 g, Vital River Laboratories, Beijing, China) were randomly divided into two groups with 12 animals each. Oral gavage of the treatments, i.e. TPT-CLNs and free TPT, inequivalent TPT doses of 5 mg/kg, were administered to the animals of every group respectively. Every six animals of each group were selected to treat more with intraperitoneal injection of 3 mg/kg cycloheximide in saline (0.6 mg/mL) as the chylomicron flow blocker before dosing with TPT-CLNs and TPT solution.

Whole blood samples were collected 0.5 mL per time point (at 0.25, 0.5, 0.75, 1, 1.5, 2, 3, 6, 8, 12, and 24 h) after oral administration. To get rid of the blood cells from plasma, final samples was obtained immediately centrifuged at 1500×*g* for 5 min after placed into centrifuge tubes with 30 μL of heparin solution (10 mg/mL) and stored frozen at −20 °C until analysis.

Preparation of plasma samples and assay procedure are as follows, mixed 100 μL plasma with 10 μL of 30% acetonitrile in water and 10 μL of 2000 ng/mL glipizide (IS solution). And then the mixed solution was added to 300 μL of ice cold methanol following the centrifugation of 10 min at 10,000×*g*. Injected 10 μL of mixed 50 μL of clear supernatant from the centrifugation and 50 μL of 1% methanoic acid onto HPLC and HPLC-MS method was established for the determination of TPT in plasma.

An Agilent 1100 HPLC system (Palo Alto, CA) consisting of a vacuum degasser, quaternary pump, and autosampler was used for solvent and sample delivery. An AB Sciex API 3000 triple-quadrupole mass spectrometer (Concord, Ontario, Canada) equipped with a turbo ion spray electrospray ionization (ESI) source was used for mass analysis and detection. Analyst version 1.4 software (AB Sciex, Concord, ON, Canada) was used for data acquisition.

Chromatographic separation was achieved using a Capcell PAK ADME cartridge column (50 × 2.1 mm, 2.7 μm, SHISEIDO) and a C_18_ guard column (4 × 3.0 mm, 5 μm, Phenomenex, Torrance, CA). The column temperature was maintained at 25 °C. The mobile phase was a mixture of methanol (A) and water containing 0.5% ethanoic acid (B). The gradient consisted of 10–100% A for 0.00–1.00 min, 100% A for 1.00–2.00 min, 100–10% A for 2.00–2.10 min, and finally decreased to 10% B to re-equilibrate the column for 4.00 min. The flow rate was 0.3 mL/min, and the injection volume was 10 μL.

The mass spectrometer was operated in the negative ion mode. Ultrapure nitrogen was used as the nebulizer, curtain, and collision-activated dissociation (CAD) gas at 8, 10, and 6 instrument units, respectively. The optimized Turbo Ion Spray voltage and temperature were set to 4500 V and 500 °C, respectively. Quantification was performed using multiple reaction monitoring (MRM) of the transitions of *m/z* 422.3 →*m/z* 377.4 for TPT, and *m/z* 446 →*m/z* 321 for the IS(GLBQ). Each transition was monitored by a 150 ms dwell time, the collision energy value and the declustering potential values were 20 and 35 V, respectively.

The plasma AUC and C_max_ of TPT was determined from this data using DAS version 2.0 software (National Aeronautics and Space Administration, Houston, TX).

#### 2.5.2. *In vivo* antitumor activity

To evaluate the antitumor activity of TPT-CLN in Male Balb/c nude mice, purchased from Beijing Vital River Laboratory Animal Technology Co., Ltd, China, more than 20 of those mice were injected subcutaneously with HEPG2 liver cancer cells. All mice were raised in a special animal room of AMMS with a standard diet and water freely available. After 10 d of the implantation, mice that successfully grew with solid tumor with a proper size (about 200 mm^3^) were randomly assigned into three experimental groups (6 per group).

Three groups were given a daily oral administration with 1 mL TPT-CLN (TPT dose of 5 mg/kg body weight), free TPT (TPT dose of 5 mg/kg body weight) and saline (control group) separately for five consecutive days (days 0–5). The observation of mice weight, tumor progression (size) and morbidity was carried out since the first day (day 0). The size (*V* = 1/6 × πab^2^, a: major diameter, b: short diameter) of subcutaneous tumor was measured by means of the vernier caliper.

The weights of tumors were measured on the last day of the experiment and those tumors were also recorded in pictures.

#### 2.5.3. TUNEL assay

The apoptotic programed cell death was detected with the method of TUNEL, which was performed using the DeadEnd™ Fluorometric TUNEL system (Promega Inc., Madison, WI). Taken from tumors of different groups, cells were washed with PBS twice for 10 min in total and fixed by 4% formaldehyde, followed by addition of 4′, 6-diamidino-2-phenylindol (DAPI). Staining was done followed the manufacturer’s instructions. Fluorescence was visualized with Olympus BX60 microscope (Olympus Optical Co., Hamburg, Germany).

#### 2.5.4. Gastrointestinal damage studies

Twelve KM male mice (AMMS, Beijing, China; 20–25 g) were divided into three groups randomly. Each of them was administrated orally with saline, free TPT and TPT-CLN at a single dose of 5 mg/kg, separately. Before the experiment, all the animals were deprived of food, but not water, for 20–24 h. Samples were obtained from standardized regions of the jejunum. Then fixed samples in neutral buffered formalin and processed by routine techniques before embedding in paraffin wax. Sections were stained by H.E dyes (Hematoxylin Eosin) and were examined under a light microscope.

### 2.6. Statistical analysis

The plasma AUC of TPT was determined using non-compartmental pharmacokinetic model with the help of Drug and Statistics Software (DAS 2.0). All the results were reported as mean ± standard deviation (SD). GraphPad Prism was used for statistical analysis. The statistical significance of calculated results among experimental groups was determined by ANOVA followed by Tukey’s test for multiple comparisons at a significance level of *p* = .05.

## 3. Results and discussion

### 3.1. Particle size and morphology analysis

**Figure 1.**
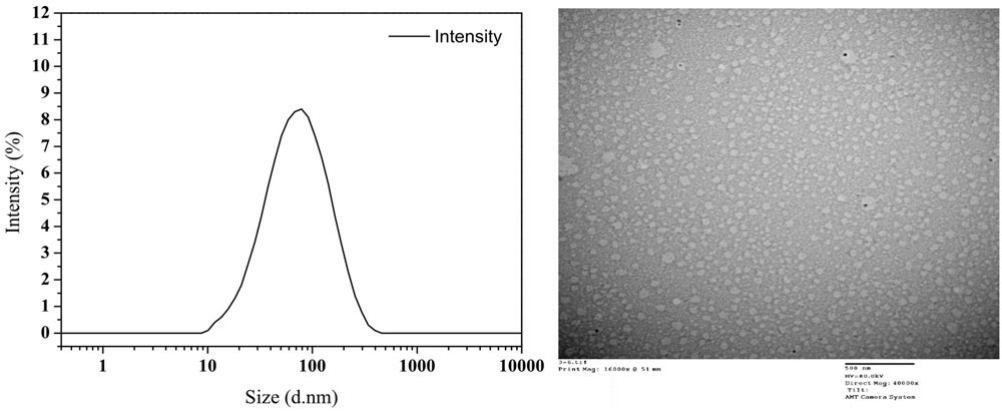
Size distribution of TPT-CLN (A) by DLS; TEM micrograph of TPT-CLN (B), the scale bar is 500 nm.

Different from simple mixture of TPT and MCT, CLN solution features a transparent appearance with light yellow color. The results of the size distribution and morphology of CLN particles are shown in the Figure 1. Particles with a spherical shape have a relatively homogeneous size distribution and most were around 60 nm.

### 3.2. *In vitro* stability

**Figure 2.**
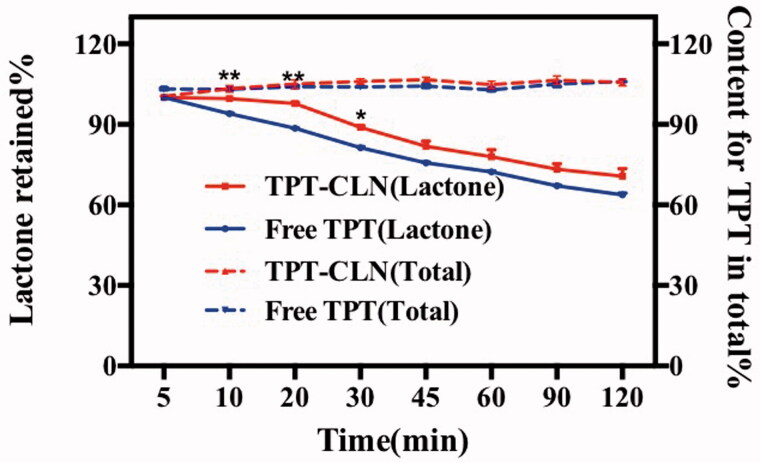
Results of *in vitro* stability at 37 °C using artificial intestinal juice as release medium (pH 6.5). Dotted line: TPT-CLN and free TPT were placed in artificial intestinal juice, the content of topotecan determined with the total of the topotecan (lactone plus carboxylate forms) as function of time; solid line: TPT-CLN and free TPT were placed in artificial intestinal juice, the content of topotecan determined with the lactone forms as function of time in artificial intestinal juice (pH 6.5). Topotecan was measured using HPLC, date point represent the mean ± SD (*n* = 3).

Figure 2 displays the *in vitro* stability of TPT at 37 °C when both TPT-CLN and free TPT solution were placed in artificial intestinal juice (pH 6.5). At every predetermined time point, more lactone form of TPT was observed from TPT-CLN than that of free aqueous solution (*p* < .01) with no difference in amount of total TPT. The results showed that TPT-CLN could significantly inhibit the intestinal hydrolytic degradation of TPT compared with free TPT by avoiding the direct contact between TPT molecules and aqueous environment.

In general, TPT is known to undergo a rapid open-ring hydrolysis and produce other more polar carboxylate with no biological activity if the pH of the aqueous solution rises above pH 4 (Burke, 1996; Giovanella et al., 2000). Therefore, once TPT releasing from normal oral formulation at physiological pH in intestinal tract, intensive hydrolytic degradation occurs inevitable and leads to poor bioavailability and anti-tumor efficacy.

On the contrary, CLN formulation exists in the form of oil droplet, TPT molecules can be protected by both phospholipid shell and oil medium barrier while avoiding the direct contact with artificial intestinal juice thus decreasing the intestinal hydrolytic degradation.

### 3.3 *In vivo* studies

#### 3.3.1. Intestinal lymphatic absorption

**Figure 3.**
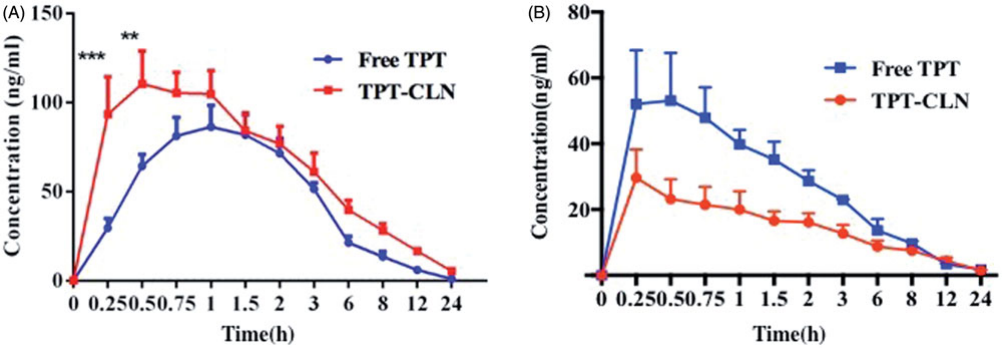
Plasma concentration versus time profiles of topotecan after oral administration of TPT-CLN or free topotecan to rats pretreated with saline (A), pretreated with 3 mg/kg cycloheximide (B) (*n* = 6 and 5 mg/kg).

The average plasma concentration profile of TPT as a function of time in the animals receiving 5 mg/kg dose equivalent treatments is shown as Figure 3. The various summarized pharmacokinetic parameters have been shown in Table 1.

**Table 1.** Pharmacokinetic parameters evaluated with topotecan concentration in rats after oral administration of two formulations pretreated with saline at a dose of 5 mg/kg.

	AUC_0–_*_*∞*_* (µg/L*h)		C_max_ (µg/L)	
Groups	Saline	Cycloheximide	Saline	Cycloheximide
Free TPT	465.041 ± 61.738**	217.398 ± 26.512*	92.125 ± 22.605**	60.297 ± 16.477**
TPT-CLN	750.447 ± 142.386**	173.173 ± 29.818*	145.014 ± 28.944**	29.633 ± 10.571**

Date presented as the mean ± SD (*n* = 6). *p< .05, **p < .01.

Figure 3 shows that the area under concentration-time curves of the group administrated with TPT-CLN was much higher than that of free TPT, when both groups without treating with cycloheximide. From Table 1, CLN enhanced the relative bioavailability of TPT by 1.62-folds versus free drug. After blocking chylomicron flow by cycloheximide, the absorption of TPT was inhibited more intensively in the animals dosed with TPT-CLN than that administrated with free TPT (*p* < .05). The AUC_0–_*_∞_* of TPT-CLN group decreased from 750.447 ± 142.386 to 173.173 ± 29.818 µg/L·h and the AUC_0–_*_∞_* of free TPT group decreased from 465.041 ± 61.738 to 217.398 ± 26.512 µg/L·h.

Pharmacokinetic parameters evaluated with TPT concentration in rats after oral administration of two formulations pretreated with saline at a dose of 5 mg/kg (Table 1).

The results described above demonstrated that CLN was able to improve the oral bioavailability of TPT by enhancing the intestinal lymphatic transportation. Therefore, when we pretreated animals with cycloheximide to block lymphatic transport using the protocol published previously (Saltz et al., 2001), the absorption of TPT in animals administrated with TPT-CLN was significantly decreased. On the contrary, for the oral administration of free TPT, the less impact of ceasing the lymphatic transport was observed. These different results demonstration CLN was expected to enhance the intestinal lymphatic absorption of the water-soluble chemotherapeutic agents, which contributing to an improvement of the oral drug bioavailability. On the other hand, it also provided a great way to obtain lymphatic targeting (Scheme 2) (Giovanella et al., 1989; Ozols, 2000).

#### 3.3.2. *In vivo* antitumor activity studies

**Figure 4.**
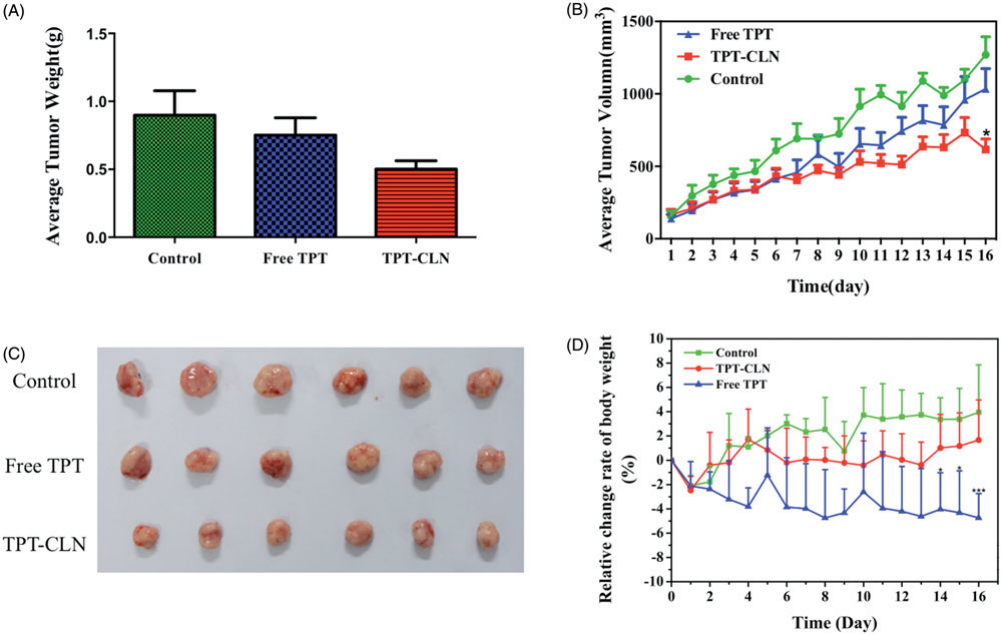
The results of *in vivo* antitumor activity studies. Tumor xenografts from HepG2 cancer cells were established subcutaneously in athymic nude mice and were treated with saline (control), TPT-CLN (500 mg/kg body weight) or free topotecan via the p.o. route for 5 consecutive days. (A) Tumor volume was measured on the 16 consecutive days, with the mean tumor volume. Data was reported as mean ± SD (*n* = 6) (*p < .05 between the free topotecan and TPT-CLN groups). (B) Measurement of tumor weight at the end of the study. (C) Photographs of excised tumor masses at the time of euthanasia on day 16 after treatment in HepG2 cancer cell xenograft-bearing mice. (D) Results of relative change rate of body weight. Data are shown as mean ± SD (*n* = 6), *p < .05, ***p < .001, Free TPT versus TPT-CLN.

The hypodermic inoculation of Balb/c nude mice, after tumors grew to a proper size (about 200 mm^3^), was followed by daily oral treatments in different experimental groups. There are three groups: control group, TPT-CLN, and free TPT. The antitumor activities of free TPT and TPT-CLN were evaluated by determining the tumor volume every day since day 0, the relative change rate of body weight, the weights of tumors at the last day, and their morphology are given in Figure 4.

As can be seen from the time-average tumor volume curve, free TPT, and TPT-CLN both showed significant antitumor activity compared to the control in the first 6 d. During the day 7∼day 16, TPT-CLN appeared to slow tumor growth more obviously than free TPT and saline (*p* < .05). On 16th day, the average tumor volumes in free TPT group and TPT-CLN group were 1034.21 and 616.42 mm^3^, respectively. From Figure 4(A), there was a significant difference between free TPT and TPT-CLN in both volume and weight (*p* < .05). These results demonstrated the TPT-CLN could improve antitumor activity of TPT in nude mice. Higher antitumor efficacy of TPT-CLN may attribute to more oral absorption of TPT as a result of enhancing lymphatic transport by CLN formulation. Furthermore, CLN could also protect TPT against the hydrolytic degradation in intestinal juice, thus more initial active form of TPT was conserved accordingly. And as a result, the more sustained and higher antitumor efficacy was obtained in presence of CLN formulation.

#### 3.3.3. TUNEL assay

**Figure 5.**
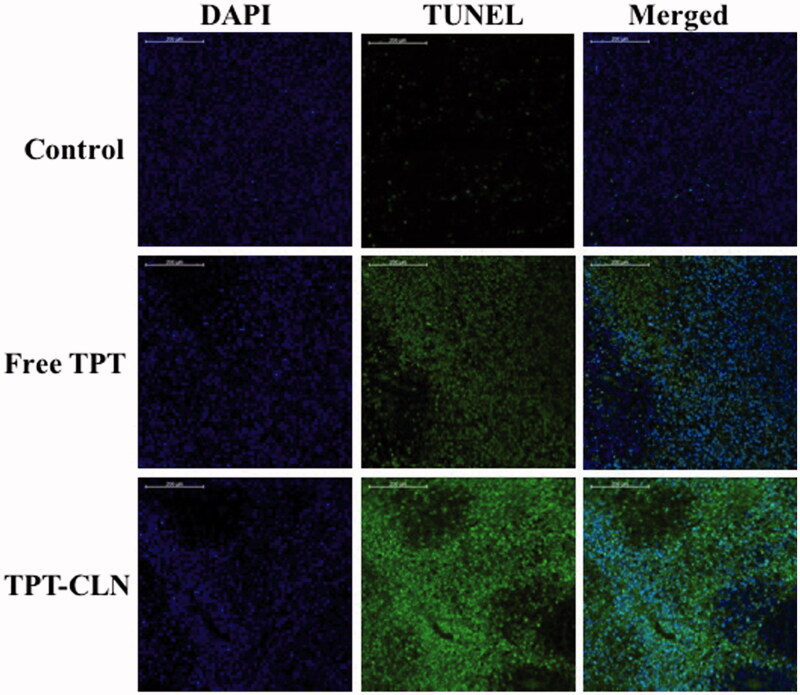
Confocal microscopy images of tumor cells from liver tumor model (nude mice) administrated with saline, free topotecan or TPT-CLN (*n* = 6, data not shown). Scale bars correspond to 200 μm.

To further compare and prove the antitumor activity, the tumors were determined by the method of TUNEL (TdT-mediated dUTP nick end labeling) that can detect DNA fragmentation resulting from apoptotic signaling cascades, originally described in the previous articles (Gavrieli et al., 1992; Lozano et al., 2009). As shown in Figure 5, few apoptotic tumor cells were observed in the control groups indicating that tumor cells were highly viable. Some apoptotic tissues were observed in the free TPT group; however, the large area of blue indicated a large number of viable tumor cells. In contrast, significant apoptosis of tumor cells was observed in the group treated with TPT-CLN, according to DAPI staining. These results were similar to antitumor activity studies in “*in vivo* antitumor activity studies”.

#### 3.3.4. Gastrointestinal damage studies

**Figure 6.**
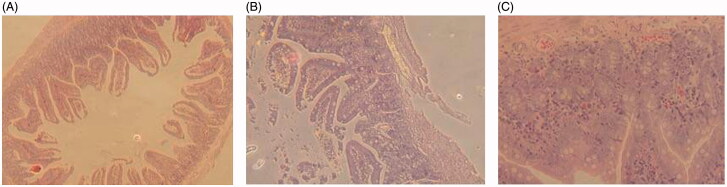
Results of gastrointestinal damage studies: light micrographs of sections of jejunum from mice treated with different treatments (*n* = 6, data not shown). (A) From a mouse in the control group that treated only with the saline. (B) From a mouse that treated with orally administration of free topotecan (5 mg/kg) for a single dose. (C) From a mouse that administrated orally with TPT-CLN at a single dose of 5 mg/kg.

All of the TPT-treated mice had extensive damage in the jejunum (Figure 6). Histologically, there was overt bleeding and necrosis throughout the jejunum with evidently extensive vascular congestion. Furthermore, parts of villi in free TPT group got widespread mucosal necrosis with cellular debris present in the lumen, whereas the other two groups had more intact villi. No histological differences were observed between TPT-CLN-treated and control group. TPT-CLN formulation could alleviate the irritation of free TPT molecules by providing both phospholipid shell and oil barrier, reducing the direct contact with intestinal mucosa in high concentration.

## 4. Conclusion

In this study, core-shell lipid (CLN) nanoparticle successfully encapsulated TPT to form oil solution. TPT molecules can be protected by both phospholipid shell and oil medium barrier while avoiding the direct contact with intestinal tract, thus decreasing the intestinal hydrolytic degradation and gastrointestinal (GI) irritation significantly. After oral administration in rats, TPT-CLN was able to improve oral absorption by enhancing the intestinal lymphatic transport comprised with free TPT. The *in vivo* antitumor activity of TPT-CLN was also investigated on tumor bearing nude mice, and the better antitumor efficacy was observed in TPT-CLN treated group contrary to free TPT. From the obtained data, the systems appeared an attractive progress in oral administration of TPT and provided a potential strategy for improving oral administration of water soluble chemotherapeutic agents.
